# Genetic Selection for Constitutively Trimerized Human HSF1 Mutants Identifies a Role for Coiled-Coil Motifs in DNA Binding

**DOI:** 10.1534/g3.113.006692

**Published:** 2013-08-01

**Authors:** Daniel W. Neef, Alex M. Jaeger, Dennis J. Thiele

**Affiliations:** Department of Pharmacology and Cancer Biology, Duke University School of Medicine, Durham, North Carolina 27710

**Keywords:** HSF1, protein chaperones, cell stress, chromatin remodeling

## Abstract

Human heat shock transcription factor 1 (HSF1) promotes the expression of stress-responsive genes and is a critical factor for the cellular protective response to proteotoxic and other stresses. In response to stress, HSF1 undergoes a transition from a repressed cytoplasmic monomer to a homotrimer, accumulates in the nucleus, binds DNA, and activates target gene transcription. Although these steps occur as sequential and highly regulated events, our understanding of the full details of the HSF1 activation pathway remains incomplete. Here we describe a genetic screen in humanized yeast that identifies constitutively trimerized HSF1 mutants. Surprisingly, constitutively trimerized HSF1 mutants do not bind to DNA *in vivo* in the absence of stress and only become DNA binding competent upon stress exposure, suggesting that an additional level of regulation beyond trimerization and nuclear localization may be required for HSF1 DNA binding. Furthermore, we identified a constitutively trimerized and nuclear-localized HSF1 mutant, HSF1 L189P, located in LZ3 of the HSF1 trimerization domain, which in response to proteotoxic stress is strongly compromised for DNA binding at the Hsp70 and Hsp25 promoters but readily binds to the interleukin-6 promoter, suggesting that HSF1 DNA binding is in part regulated in a locus-dependent manner, perhaps via promoter-specific differences in chromatin architecture. Furthermore, these results implicate the LZ3 region of the HSF1 trimerization domain in a function beyond its canonical role in HSF1 trimerization.

All organisms are exposed to stressful conditions that result in the accumulation of misfolded and aggregation-prone proteins that, unless appropriately managed, result in cell dysfunction and death. To respond to these stresses, cells have evolved adaptive mechanisms to protect and stabilize cellular proteins until more favorable conditions for cellular proliferation are encountered. The heat shock transcription factor, HSF, is a conserved, homotrimeric transcription factor that activates gene expression in response to a variety of stresses, including heat shock, oxidative stress, as well as inflammation and infection (Akerfelt *et al.* 2010; Bjork and Sistonen 2010; Anckar and Sistonen 2011). Among HSF target genes are those encoding protein chaperones, which assist in protein folding and protect from stress-induced cell death, and other genes encoding proteins with many distinct functions in cellular homeostasis (Hartl and Hayer-Hartl 2002; Hahn *et al.* 2004; Trinklein *et al.* 2004; Gonsalves *et al.* 2011). Recent evidence has shown that in *Saccharomyces cerevisiae* HSF directly activates the expression of genes whose protein products are involved in protein folding and degradation, ion transport, signal transduction, energy generation, carbohydrate metabolism, vesicular transport, cytoskeleton formation, and a broad array of other cellular functions (Hahn *et al.* 2004). Collectively the stress-dependent activation of target gene expression by HSF is known as the heat shock response.

In *S. cerevisiae* the heat shock response is mediated by a single HSF that is essential for cell viability under all conditions evaluated (Sorger and Pelham 1988). Although mammalian cells express four nonessential HSF proteins encoded by separate genes, HSF1 is the primary mammalian heat shock factor responsible for stress responsive gene transcription (Akerfelt *et al.* 2010), with HSF2 also modestly activating protein chaperone gene expression under less acute temperature conditions (Fujimoto and Nakai 2010; Shinkawa *et al.* 2011). In the absence of proteotoxic stress the activity of mammalian HSF1 is repressed through a variety of mechanisms that are not fully understood. HSF1 is bound and repressed by the protein chaperones Hsp90 and Hsp70, though the mechanisms for how these chaperones repress HSF1 activity remain unclear (Abravaya *et al.* 1992; Baler *et al.* 1996; Shi *et al.* 1998; Zou *et al.* 1998). It is hypothesized that during the initial response to proteotoxic stress, the inactive cytosolic HSF1 monomer dissociates from Hsp90, forms a homotrimer which is transported to the nucleus to bind to heat shock elements (HSE) found in the promoters of HSF1 target genes and promotes gene activation (Sarge *et al.* 1993; Cotto *et al.* 1996; Xia and Voellmy 1997; Zou *et al.* 1998). In response to stress, HSF1 also undergoes several post-translational modifications including sumoylation and hyper-phosphorylation (Sarge *et al.* 1993; Cotto *et al.* 1996; Xia and Voellmy 1997; Hietakangas *et al.* 2003).

HSF1 is also thought be maintained in an inactive monomeric state through an intramolecular coiled-coil interaction between a leucine zipper (LZ4) in the carboxyl-terminus of the protein and three leucine zippers (LZ1-3) in the amino-terminus, that are also required for homotrimerization during stress activation (Figure 1A) (Sorger and Nelson 1989; Rabindran *et al.* 1993; Zuo *et al.* 1994, 1995). The individual helices of a typical coiled-coil domain contain repeats of seven amino acid arrays consisting of hydrophobic and charged amino acid residues which arrange themselves in such a fashion that the hydrophobic interactions among the helices provide the thermodynamic force for oligomerization, in part guided by the ionic interactions across heptad repeats (Sodek *et al.* 1972). Although the interaction between LZ4 and the trimerization domain (LZ1−3) of HSF1 is hypothetical and has not yet been described experimentally, this hypothesis suggests a model in which HSF1 exists in an equilibrium between an active trimeric state, mediated by coiled-coil interactions of the trimerization domain, and an inactive state, mediated by coiled-coil interactions between the trimerization domain and LZ4 (Figure 1A). Whether these coiled-coil domains play other roles in the regulation of HSF1 is not known.

**Figure 1:**
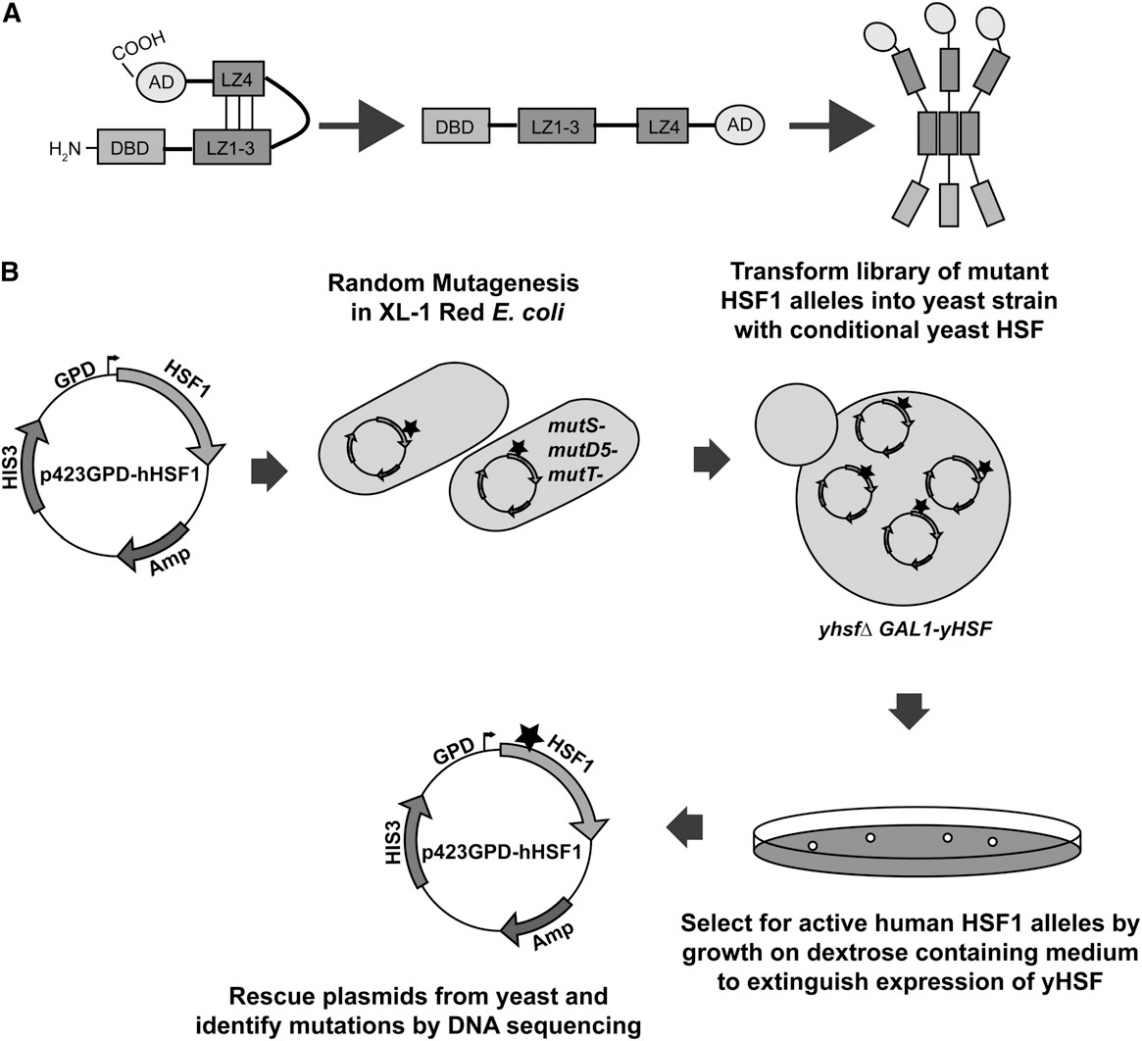
(A) Leucine zippers are thought to repress HSF1 activity and facilitate HSF1 homotrimerization via coiled-coil interactions. (B) Screen used to identify constitutively trimerized alleles of human HSF1. A yeast plasmid expressing human HSF1 was transformed into mutagenic XL-1 Red *E. coli* to generate a library of mutant HSF1 plasmids that was transformed into a yeast strain with a conditionally expressed yeast HSF allele. Constitutively trimerized human HSF1 alleles were selected by growth on dextrose containing medium, which extinguishes expression of yeast HSF, and identified by DNA sequencing.

Despite a high level of conservation of both HSF1 and the cognate HSEs from yeast to humans, our previous results demonstrated that expression of the human HSF1 is unable to complement for the loss of the essential yeast HSF (Liu *et al.* 1997). This inability to complement appears to be predominantly attributable to an inability of human HSF1 to form a homotrimer in yeast, an essential part of the activation process. Consequently, human HSF1 is unable to bind to and activate HSE-dependent gene expression to support yeast viability. However, the expression of a human HSF1 mutant with amino acid substitutions in LZ4, which is constitutively trimerized in mammalian cells, can function in *S. cerevisiae* (Liu *et al.* 1997). Further studies in yeast also identified an amino-terminal linker-domain, a loop in the DNA binding domain, as well as several phosphorylation sites as repressive elements that contributed to HSF1 repression in both yeast and mammalian cells (Liu and Thiele 1999; Ahn *et al.* 2001; Batista-Nascimento *et al.* 2011). Together, these results suggest that when human HSF1 is expressed in yeast, it is maintained in a constitutively repressed monomeric state through mechanisms that are similar to those of mammalian cells and that the yeast system can serve as a simplified assay system to decipher aspects of the complex mechanisms regulating human HSF1 activity in mammalian cells.

Here we report the use of the humanized yeast assay system to further understand the mechanisms that regulate human HSF1 through a random mutagenesis screen. We identify novel human HSF1 mutants in the leucine zippers of HSF1 which result in the constitutive trimerization and nuclear localization of HSF1. Interestingly, our results reveal that despite their constitutively trimerized state, the HSF1 mutants we identified are unable to bind DNA in the absence of stress in mammalian cells, suggesting that additional stress responsive events are required to elicit HSF1 DNA binding. Furthermore, we report the identification of a constitutively trimerized HSF1 mutant exhibiting genomic locus-specific DNA binding defects *in vivo*.

## Materials and Methods

### Random mutagenesis screen and yeast cell growth

A library of human HSF1 mutants was generated by transforming the plasmid pRS423GPD-hHSF1 (Batista-Nascimento *et al.* 2011) into the *Escherichia coli* XL-1 Red strain deficient in three primary DNA repair pathways (*mutS*, *mutD*, *mutT*) resulting in a spontaneous mutagenesis rate ~5000-fold greater than wild-type cells. Plasmid transformed bacterial colonies were scraped off plates, pooled, and inoculated into liquid growth medium for plasmid maxiprep. The resulting library of mutant HSF1 plasmids were transformed into the previously described yeast strain PS145 (*MAT* **a** *ade2-1 trp1-1 can1-100 leu2-3,112 his3-11,15 ura3-1 hsf1*Δ::*LEU2* pGAL-*HSF*:*URA3*) (Liu *et al.* 1997), and yeast cells were plated on medium containing 2% dextrose to extinguish expression of yeast HSF and select for those human HSF1 mutants able to promote human HSF1-dependent yeast growth. Plasmids were rescued from the resulting yeast colonies, re-transformed to confirm activity and HSF1 sequence analyzed by DNA sequencing. Yeast strain PS145, transformed with either a wild-type or mutant HSF1 expressing plasmid, was analyzed in liquid growth curve assays and spot assays as previously described (Batista-Nascimento *et al.* 2011).

### Immunobloting, trimerization, and subcellular localization experiments

Total protein was extracted from 1 × 10^8^ yeast cells by incubation in 200 µL of NaOH lysis buffer (0.1 M NaOH, 0.05 M ethylenediaminetetraacetic acid [EDTA], 2% sodium dodecyl sulfate [SDS], 2% beta-mercaptoethanol) for 10 min at 90°. The NaOH was neutralized by the addition of 5 µL of 4 M acetic acid and after the addition of Laemmli buffer proteins were fractionated by SDS polyacrylamide gel electrophoresis and analyzed by immunoblotting using anti-HSF1 (Neef *et al.* 2010) and anti-PGK1 (22C5D8; Abcam) antibodies.

For protein analysis from mammalian cells wild-type and mutant HSF1-expressing plasmids were transfected into hsf1^−/−^ mouse embryonic fibroblasts (MEF), previously described (McMillan *et al.* 1998), using a 4D Nucleofector (Lonza). Mammalian protein extracts were prepared and assayed for expression of Hsp70 by immunoblotting and enzyme-linked immunoassay (ELISA) as previously described (Neef *et al.* 2010). The antibodies used in this study were anti-Hsp70 (W27; Santa Cruz Biotechnology) and anti-GAPDH (6C5; Santa Cruz Biotechnology). Analysis of HSF1 trimerization and cellular localization were performed as previously described (Neef *et al.* 2010). Indirect immunofluorescence utilizing an Axio Imager was performed as described (Nevitt and Thiele 2011) using an anti-HSF1 antibody (10H8, Enzo)

### DNA binding studies

MEF cells transfected with wild-type or mutant HSF1 proteins were heat shocked at 42° for 20 min followed by crosslinking via the addition of 1% formaldehyde for 5 min on ice. The formaldehyde crosslinker was quenched on ice by the addition of 375 mM glycine for 5 min. The cross-linked cells were washed twice in phosphate-buffered saline, harvested, and suspended in cell lysis buffer (25 mM Tris, 150 mM NaCl, 1 mM EDTA, 1% Triton X-100, 0.1% SDS) supplemented with protease inhibitors. Cell lysates were diluted twofold by the addition of IP Buffer (50 mM Tris, 150 mM NaCl, 1 mM Triton X-100), and the chromatin was sheered by sonication three times for 30 sec using a Sonic Dismembrator 550 on setting 3 and then incubated with 5 µL of anti-HSF1 antibody (Neef *et al.* 2010) for 18 hr at 4°. Protein−antibody complexes were purified using 50 µL of Protein A agarose beads for 3 hr at 4° and washed twice in IP-buffer, twice in IP-buffer + 0.5 M NaCl, and twice in 1X TE buffer at 24°. Protein−DNA complexes were eluted from beads by incubating in TES (50 mM Tris, 10 mM EDTA, 1% SDS) at 65° for 10 min and crosslinks were reversed overnight at 65°. The purified proteins were digested by the addition of Proteinase K, and the DNA was purified using the GFX PCR DNA and Gel Purification kit (GE Healthcare). DNA binding of HSF1 was assessed at the Hsp70, Hsp25, and interleukin (IL)-6 promoters as well as the GAPDH open reading frame (negative control) by qPCR using the following primers: Hsp70, forward CACCAGCACGTTCCCCA, Hsp70 reverse CGCCCTGCGCCTTTAAG; Hsp25, forward CCGTCATTTGTTTTCTTCAACAAG, Hsp25 reverse GCACCCCGAAAGCTTGATC; IL-6, forward GCAACTCTCACAGAGACTAAAGG, IL-6 reverse GGACAACAGACAGTAATGTTGC; GAPDH forward AGAGAGGGAGGAGGGGAAATG, GAPDH reverse AACAGGGAGGAGCAGAGAGCAC.

## Results

### Yeast screen for constitutively active HSF1 mutants

Our previous studies demonstrated that expression of the human HSF1 protein is unable to complement for the loss of the essential yeast HSF due to an inability to form a homotrimer, even in response to proteotoxic stress conditions such as heat shock (Liu *et al.* 1997). However, mutations in the LZ4 coiled-coil domain, or in repressive phosphorylation sites that render human HSF1 constitutively trimerized in mammalian cells, promote constitutive activation of human HSF1 in yeast and allow for complementation (Liu *et al.* 1997; Batista-Nascimento *et al.* 2011). Together, these data suggest that when human HSF1 is expressed in yeast the protein is maintained in a repressed monomeric state that is unable to respond to proteotoxic stresses and that mutations that enhance the propensity for trimer formation may overcome this repression. This conservation of regulatory mechanisms when human HSF1 is expressed in yeast allowed for the development of a facile assay system to identify human HSF1 mutants that allow human HSF1 complementation in yeast.

Our screen, outlined in Figure 1B, used a library of human HSF1 mutant clones generated using the mutagenic XL1-Red *E. coli* strain. The mutant HSF1 library was transformed into yeast strain PS145, which lacks the genomic copy of yeast HSF and expresses yeast HSF from a plasmid under the control of the galactose-inducible and dextrose-repressible *GAL1-10* promoter. After transforming the mutant HSF1 library into yeast strain PS145, recipient cells were selected for mutant human HSF1 clones able to complement for the loss of yeast HSF by growth on dextrose containing medium, which extinguishes expression of yeast HSF and render yeast growth solely dependent on human HSF1 function.

**Figure 2:**
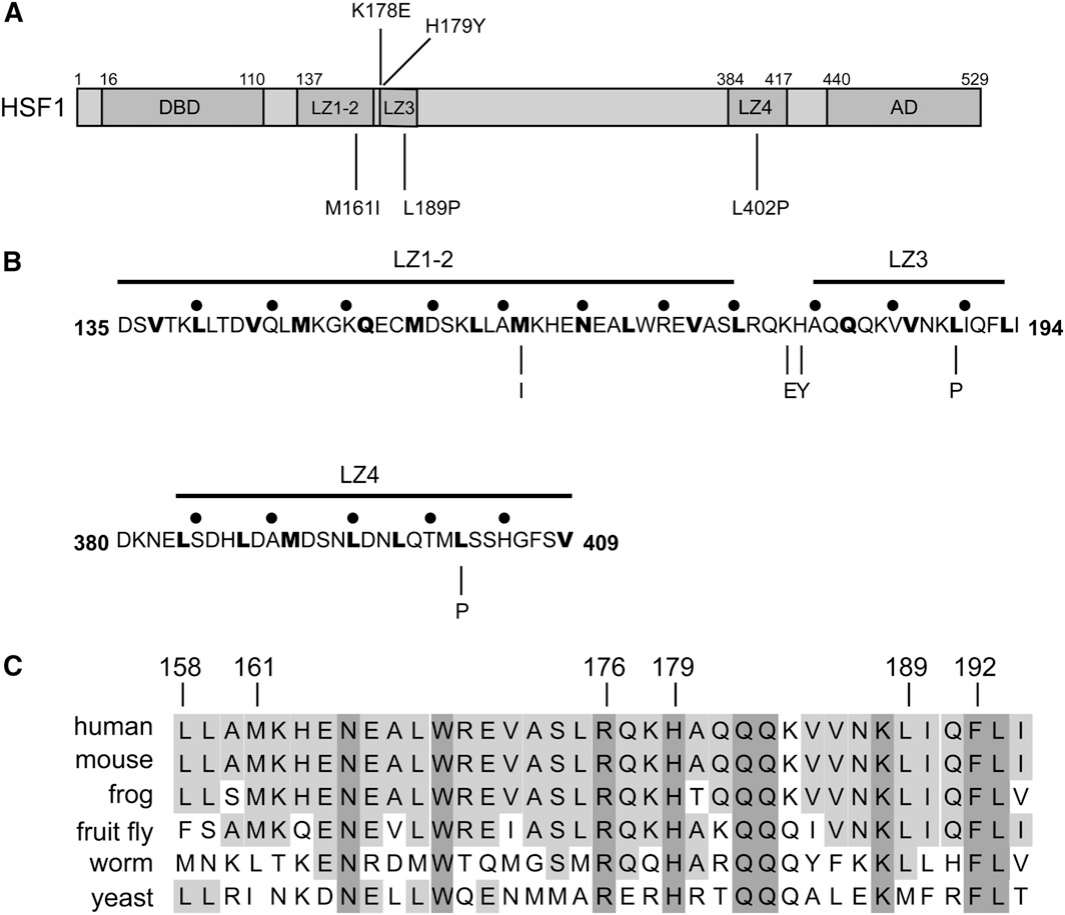
Constitutively trimerized human HSF1 alleles have mutations in leucine zippers. (A) Schematic showing the location of mutations identified in this study that promote human HSF1-dependent yeast growth. (B) Mutations that promote human HSF1 dependent yeast growth mutate key residues in leucine zipper domains. For reference, black dots are placed at five amino acid intervals. (C) Mutations that promote human HSF1 dependent yeast growth occur in highly conserved amino acid residues.

Sequence analysis of those human HSF1 alleles able to complement for the loss of yeast HSF revealed five distinct mutations (Figure 2A). Although four of the five mutations identified were located in the three leucine zipper domains (LZ1-3) within the trimerization domain of HSF1 (Figure 2A) one mutation was located in the carboxyl-terminal leucine zipper domain (LZ4), a finding that corresponds to the known repressive nature of this domain. Specifically, double mutations in LZ4 (M391K and L395P) were previously described by Rabindran *et al.* (1993) to promote the constitutive trimerization of human HSF1 in Hek293 cells and triple mutations in LZ4 (M391K, L395P, L398R) have been shown by our laboratory to promote the activation of human HSF1 in yeast (Liu *et al.* 1997). The results presented here demonstrate that a single amino acid substitution in a key hydrophobic residue (L402P) of LZ4 can also promote trimerization of human HSF1 and allow for complementation of human HSF1 in yeast.

**Figure 3:**
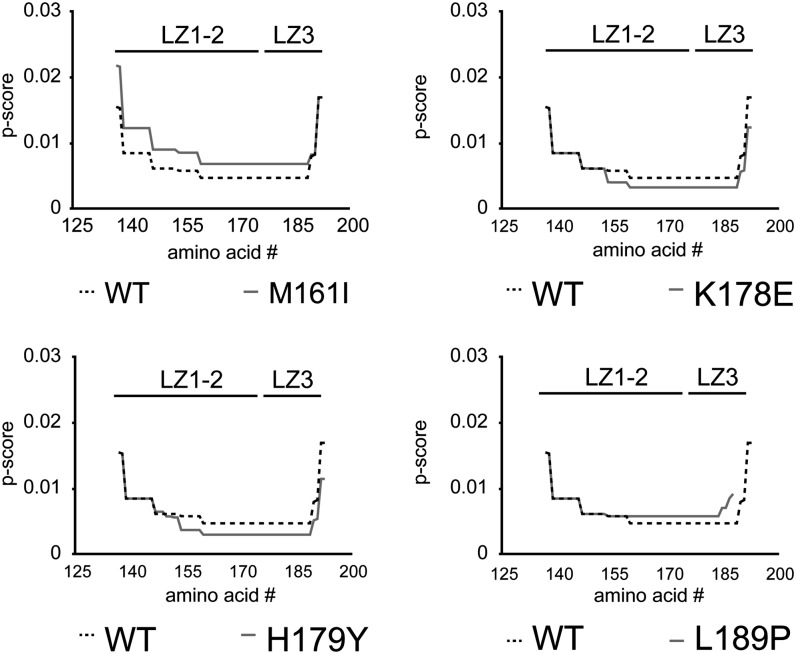
Mutations in HSF1 leucine zippers modulate coiled-coil propensity. Coiled-coil propensity for the wild-type and mutant LZ1-3 (a.a. 137-193) was analyzed via the Paricoil2 algorithm (http://groups.csail.mit.edu/cb/paircoil2/). The data are represented as p-score as a function of amino acid residues. The approximate location of LZ1-2 and LZ3 is represented above each graph. p-score < 0.02 constitutes a coiled-coil domain.

Two of the human HSF1 mutants we identified in LZ1-3 (M161I, L189P) as well as the mutant we identified in LZ4 (L402P) occur at key hydrophobic residues (Figure 2B, bold), and are conserved across a variety of species from yeast to humans (Figure 2C). Thus, we predict that these residues are potentially required for the formation of the predicted repressive coiled-coil interactions (Figure 1A). However, it is of interest that the isoleucine substitution for Met161 results in the replacement of a hydrophobic amino acid with another hydrophobic amino acid. Thus, we used the Paircoil2 algorithm (McDonnell *et al.* 2006) to analyze the effects of this and the other mutations we identified in LZ1-3 on the propensity for coiled-coil formation. Interestingly, despite its hydrophobic nature, substitution of M161 with isoleucine is predicted to reduce the propensity for coiled-coil formation (Figure 3). Furthermore, we also identified proline substitutions in both LZ1-3 and LZ4 (L189P, L402P), which are well known to disrupt alpha helices and are likely to have detrimental effects on coiled-coil formation. As predicted by the Paircoil2 algorithm, the proline substitution of L189 strongly reduces the propensity for coiled-coil formation (Figure 3). However, it is important to note that the entire trimerization domain is not predicted to be eliminated by this substitution, but rather only a small portion of the coiled-coil domain adjacent to the proline substitution which corresponds to LZ3, is predicted to be perturbed. Interestingly, LZ3 has previously been linked to HSF1 repression (Zuo *et al.* 1994, 1995).

We also identified mutations in two charged amino acids located between LZ1/2 and LZ3 (K178E, H179Y) which are predicted by the Paircoil2 algorithm to increase the propensity for coiled-coil formation of the trimerization domain and may thereby shift the equilibrium of HSF1 from a monomeric to homotrimeric state (Figure 3). As the tyrosine substitution of His179 substitutes a hydrophobic amino acid for a charged amino acid, it is possible that this mutation simply extends the LZ1/2 domain further and increases the size of the trimerization domain and is consistent with a previous study that identified an arginine substitution of His179 that also promoted constitutive trimerization of HSF1 (Farkas *et al.* 1998). However, it remains unclear how the charged glutamate substitution of Lys178 increases coiled-coil propensity.

### Mutations in HSF1 promote constitutive trimerization and complementation in yeast

**Figure 4:**
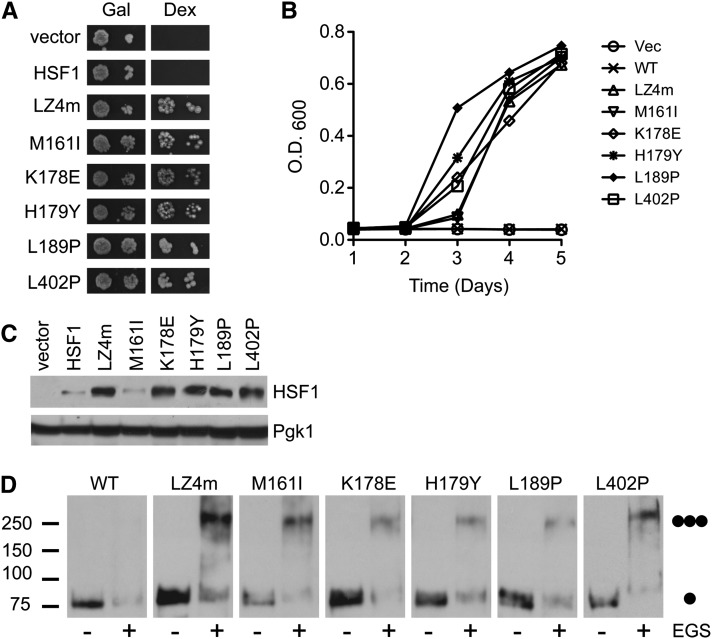
Leucine zipper mutations promote human HSF1−dependent yeast growth and promote constitutive trimerization of human HSF1 in yeast. (A) PS145 yeast strains expressing wild-type human HSF1 or the HSF1 mutants identified in this screen were plated on either galactose or dextrose supplemented medium. The previously described LZ4m mutant allele has three mutations in LZ4 and serves as a positive control. (B) PS145 expressing either wild-type human HSF1 or the HSF1 mutants identified in this screen were grown in dextrose containing medium for 4 d. Growth was monitored by measuring the optical density of a sample measured at a wavelength of 600 nm (OD_600_). (C) PS145 was transformed with a plasmid expressing wild-type human HSF1 or mutant alleles of HSF1 and grown on galactose containing medium. Total protein extracts were analyzed for HSF1 and Pgk1 by immunoblotting. (D) PS145 was transformed with wild-type human HSF1, the LZ4m mutant or the HSF1 mutants identified in this screen and grown on galactose containing medium. Total protein extracts were evaluated for HSF1 multimerization by EGS crosslinking, SDS polyacrylamide gel electrophoresis, and immunoblotting using an HSF1-specific antibody. The positions of molecular weight markers are indicated on the left, and circles indicating the expected migration of HSF1 monomers and trimers are on the right.

The ability of the HSF1 mutants we identified to promote human HSF1-dependent yeast growth was confirmed by a qualitative spot assay analysis (Figure 4A) as well as quantitative growth curve analysis (Figure 4B). More quantitative growth curve analysis revealed that although all of the human HSF1 mutants identified promoted yeast growth, the HSF1 L189P mutant was somewhat more potent at activating yeast growth in comparison to the other mutants we identified. The remaining mutants displayed essentially similar efficacy in their ability to promote yeast growth in this assay (Figure 4B).

We previously showed that constitutively trimerized human HSF1 mutants are detected at higher steady state levels in comparison to wild-type human HSF1 in yeast (Batista-Nascimento *et al.* 2011). In accordance with these results, all of the human HSF1 mutants identified here except for the M161I mutant were detected at increased steady state levels when compared to the wild-type protein (Figure 4C), indicating that an elevation in human HSF1 steady state protein levels is not required to complement for loss of yeast HSF. As predicted by our previous findings, the ability of HSF1 mutants we identified to promote yeast growth should be dependent on their ability to homotrimerize. In support of this hypothesis, EGS crosslinking experiments reveal that while wild-type HSF1 exists predominantly in the monomeric form, the active HSF1 mutants identified were all enriched in the trimer form (Figure 4D).

### Differential activation of gene expression by HSF1 mutants in mammalian cells

**Figure 5:**
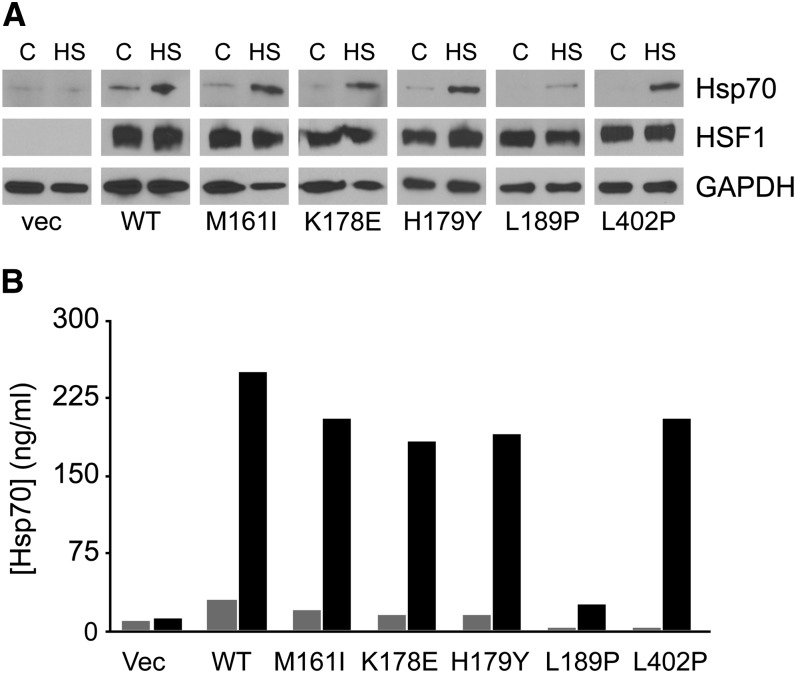
The L189P HSF1 mutant is defective in promoting Hsp70 expression in mammalian cells. (A) hsf1^−/−^ MEFs were transiently transfected with an empty vector or plasmids expressing wild-type human HSF1 or the HSF1 mutants identified in this screen. The transfected cells were maintained either at 37° or heat shocked at 41° for 1 hr followed by an 8-hr recovery at 37°. Total protein extracts were analyzed for Hsp70 and HSF1 by immunoblotting. GAPDH serves as a loading control. (B) Cell lysates were prepared as in (A) and analyzed for Hsp70 levels by ELISA.

To test how the human HSF1 mutants identified in yeast function in mammalian cells, wild-type or mutant HSF1 alleles were transiently transfected into hsf1^−/−^ MEFs and their ability to promote stress-responsive transactivation of gene expression was assessed by assaying Hsp70 protein expression by both immunoblotting and ELISA experiments. Consistent with the notion that regulation of HSF1 *trans*-activation is a complex process involving homotrimerization, nuclear localization, DNA binding and stress-responsive hyperphosphorylation, none of the HSF1 mutants tested promoted Hsp70 expression in the absence of stress beyond that observed by wild type HSF1 (Figure 5, A and B). Interestingly, the HSF1 L189P mutant, which was constitutively trimerized and functional in yeast cells, was strongly compromised in its ability to promote HSF1-dependent Hsp70 expression under stress conditions (Figure 5, A and B).

**Figure 6:**
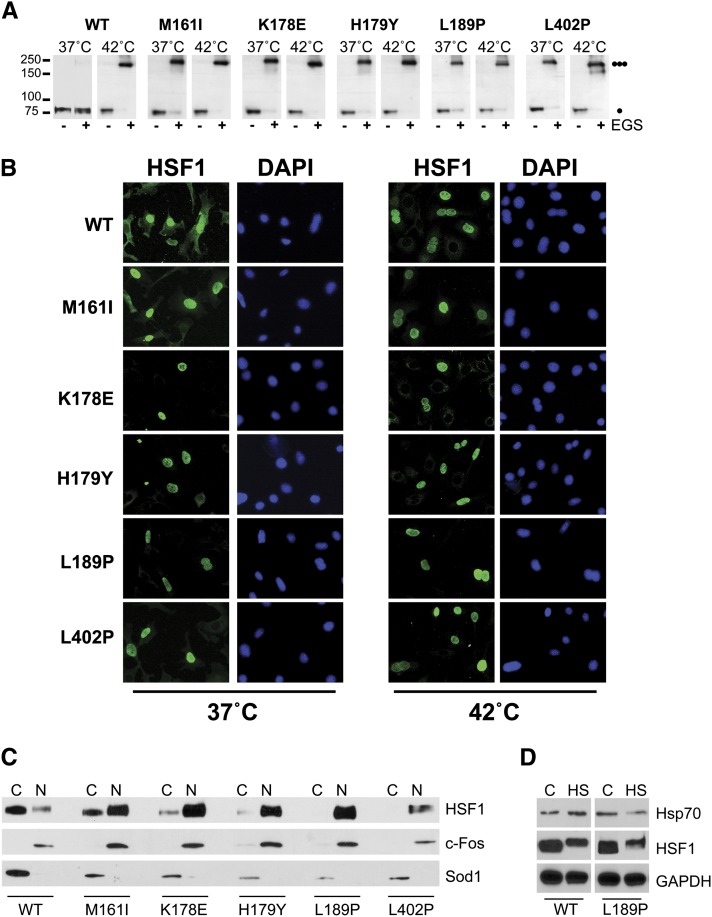
HSF1 leucine zipper mutations promote constitutive trimerization and nuclear localization in mammalian cells. (A) hsf1^−/−^ MEFs were transfected with a plasmid expressing wild-type HSF1 or the HSF1 mutants identified in this screen and analyzed for HSF1 multimerization by EGS cross-linking. The positions of molecular weight markers are indicated on the left, and circles indicating the expected migration of HSF1 monomers and trimers are on the right. (B) hsf1^−/−^ MEFs were transfected with a plasmid expressing wild-type HSF1 or the HSF1 mutants identified in this screen and assayed for localization by immunofluorescence. (C) hsf1^−/−^ MEFs were transfected with a plasmid expressing wild-type HSF1 or the HSF1 mutants identified in this screen and nuclear and cytoplasmic fractions were analyzed for HSF1 by immunoblotting. c-fos and SOD1 serve as nuclear (N) and cytoplasmic (C) markers, respectively. (D) hsf1^−/−^ MEFs were transfected with a plasmid expressing wild-type HSF1 or the L189P HSF1 mutant, heat shocked at 42° for 30 min and protein extracts were assayed for HSF1 hyper-phosphorylation by immunoblotting for an HSF1 mobility shift. Total protein extracts were also immunoblotted for Hsp70 and GAPDH serves as a loading control.

Because the L189P HSF1 mutant was compromised in its ability to promote Hsp70 expression in mammalian cells, we ascertained if the human HSF1 mutants we identified in our yeast screen were also constitutively trimerized in mammalian cells. In correlation with the results obtained in yeast, EGS crosslinking experiments in mammalian cells revealed that in the absence of stress, wild-type HSF1 exists predominantly as a monomer, which is converted to the trimeric form in response to heat stress. In contrast, each of the HSF1 mutants exist predominantly in the trimeric form and stress exposure only modestly enhanced trimerization (Figure 6A). In previous experiments authors have shown that mammalian HSF1 trimerization is a pre-requisite for nuclear accumulation (Wu 1995). Therefore, we hypothesized that these mutants should also be localized to the nucleus even in the absence of stress. This hypothesis was supported by indirect immunofluorescence microscopy and cell-fractionation experiments which demonstrated that while the wild-type protein exists predominantly in the cytoplasm, the mutants we identified are localized to the nucleus in the absence of stress and remained nuclear in response to a heat shock (Figure 6, B and C).

Our observation that the L189P HSF1 mutant is constitutively trimerized and constitutively localized to the nucleus, but is deficient in its ability to promote heat shock-dependent Hsp70 expression suggests that this mutant is defective in an important aspect of the HSF1 activation pathway downstream of nuclear localization. Although a definitive role for HSF1 hyperphosphorylation has not been elucidated, it is well established that HSF1 is hyperphosphorylated as part of the stress-dependent HSF1 activation pathway. Our data show that, similar to the wild-type HSF1 protein, the L189P mutant is readily hyperphosphorylated in response to heat stress-exposure and suggest that a different aspect of the HSF1 activation pathway is affected by the L189P mutation (Figure 6D).

### Constitutively trimerized HSF1 mutants are not constitutively bound to DNA

On the basis of conventional model for the mammalian HSF1 activation pathway, once HSF1 is trimerized and accumulates in the nucleus, it would be competent for DNA binding to promoter HSEs (Sarge *et al.* 1993; Zuo *et al.* 1994, 1995; Cotto *et al.* 1996). Because the human HSF1 mutants isolated in this study are constitutively trimerized and nuclear-localized we hypothesized that these mutants should be constitutively bound to DNA *in vivo*. To address this hypothesis we performed chromatin immunoprecipitation experiments using MEF cells transfected with plasmids expressing wild-type HSF1 or the HSF1 mutants we identified in this study.

**Figure 7:**
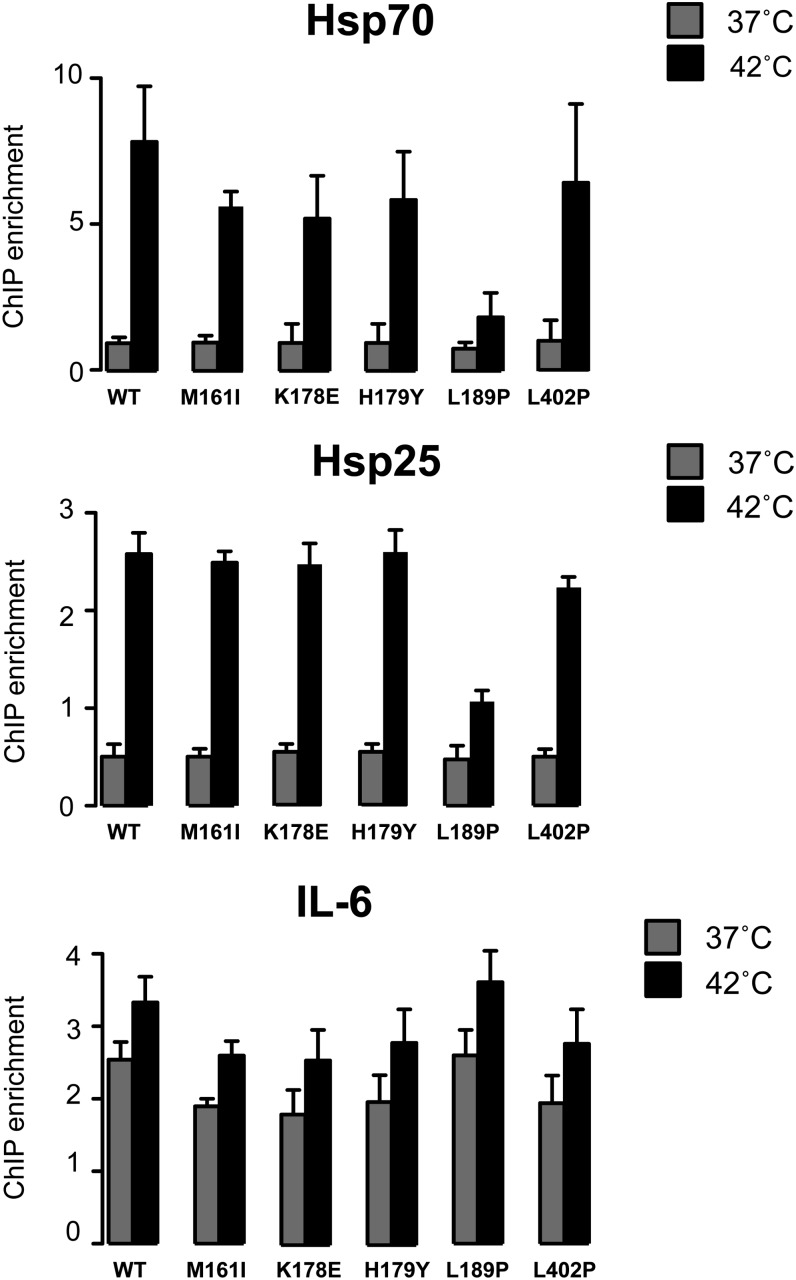
The L189P HSF1 mutant exhibits genomic locus dependent deficiencies in DNA binding. hsf1^−/−^ MEFs were transfected with a plasmid expressing wild-type HSF1 or the HSF1 mutants identified in this screen and analyzed for HSF1 DNA binding by chromatin immunoprecipitation at the Hsp70, Hsp25 and IL-6 promoters under normal growth conditions (37°) or 1 hr heat shock at 42°.

Because previous *in vitro* DNA binding studies have shown that constitutively trimerized HSF1 mutants readily bind DNA (Rabindran *et al.* 1993; Zuo *et al.* 1994, 1995; Farkas *et al.* 1998), it was surprising and contradictory to the current model of the HSF1 activation pathway to find that the constitutively trimerized HSF1 mutants did not bind to the Hsp70 or Hsp25 promoters in the absence of stress beyond that observed for the monomeric wild-type human HSF1 (Figure 7). These observations suggest that the ability of HSF1 to bind DNA *in vivo* is more complex than what is observed *in vitro* and may not be solely dependent on HSF1 trimer formation, perhaps requiring additional stress-responsive factors. However, upon exposure to heat stress, inducible binding to the Hsp70 and Hsp25 promoters was observed for wild-type HSF1 as well as most of the mutant HSF1 proteins (Figure 7). Interestingly the HSF1 L189P mutant was strongly compromised for binding to both the Hsp70 and Hsp25 promoters even under stress conditions, consistent with the compromised ability of this mutant protein to induce Hsp70 expression.

The reduced ability of the HSF1 L189P mutant protein to bind to promoter DNA *in vivo* and promote Hsp70 expression even in response to heat stress was surprising, as the HSF1 trimerization domain has not previously been linked to DNA binding. To further evaluate the DNA binding propensity of these mutant proteins, we assayed HSF1 DNA binding at the IL-6 promoter where HSF1 has previously been shown to bind constitutively, even in the absence of stress (Takii *et al.* 2010). In concert with previously published findings, wild-type HSF1 was bound to the IL-6 promoter in the absence of stress and binding increased modestly upon exposure to heat shock (Figure 7). Similar levels of HSF1 DNA binding were observed for all the HSF1 mutants including the L189P mutant, suggesting that this mutation does not directly impinge on the function of the HSF1 DNA binding domain but rather imparts a genomic locus specific DNA binding defect, perhaps by precluding the interaction with auxiliary factors that are required for modulating loci-specific chromatin landscapes.

### The L189P HSF1 mutant fails to protect from heat stress−induced cytotoxicity

**Figure 8:**
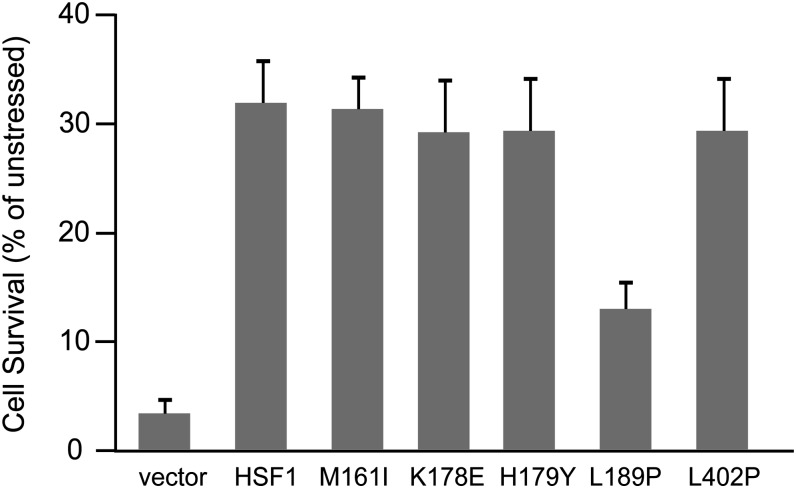
The L189P HSF1 mutant exhibits deficiencies in protecting cells from thermal stress. hsf1^−/−^ MEFs transfected with a plasmid expressing wild-type HSF1 or the HSF1 mutants identified in this screen were heat shocked at 42° for 2 hr followed by an 18-hr recovery. Cell viability was assayed by Cell Titer Glo assay.

The inability of the HSF1 L189P mutant to bind DNA and promote heat shock-dependent Hsp70 or Hsp25 expression *in vivo* suggested that cells expressing this mutant isoform of HSF1 might be severely compromised in their ability to protect from cytotoxic stress. To test this hypothesis we assayed the viability of hsf1^−/−^ MEFs transfected with either an empty vector, wild-type HSF1 or the M161I, K178E, H179Y, L189P or L402P HSF1 mutants after exposure to a 42° heat shock for 2 h. The results of these experiments show that although the wild-type HSF1 as well as the M161I, K178E, H179Y and L402P HSF1 proteins were able to protect cells from heat stress-induced cell death, the HSF1 L189P mutant was strongly compromised in its ability to provide cytoprotection (Figure 8). However, the protection from cytotoxic stress achieved by the L189P HSF1 mutant is greater than what we observed for cells transfected with an empty vector, consistent with observations presented here that this mutant retains a low level of DNA binding activity (Figure 7) and does promote low levels of Hsp70 expression in response to heat shock (Figure 5, A and B).

## Discussion

Many studies during the past two decades have demonstrated that the activation of mammalian HSF1 is a complex, multistep process involving the conversion of an inactive cytosolic monomer to a homotrimer, nuclear localization, DNA binding, and gene *trans*-activation (Sarge *et al.* 1993; Zuo *et al.* 1994, 1995; Cotto *et al.* 1996) as sequential and highly regulated events. When human HSF1 is expressed in yeast, its ability to homotrimerize, bind DNA and promote gene activation is repressed (Liu *et al.* 1997) suggesting that the mechanisms regulating HSF1 activity are at least partially conserved between yeast and mammalian cells. Moreover, consistent with this notion, we previously demonstrated that small molecules that activate human HSF1 in yeast cells also activate HSF1 in cultured mammalian cells and in fruit flies (Neef *et al.* 2010). We have used this repression of human HSF1 trimerization in yeast to create and utilize a screen aimed at identifying novel mutations that promote the constitutive trimerization of human HSF1 as a means to further decipher this complex regulatory pathway. Here we report the identification of mutations that lead to constitutive trimerization of human HSF1 in yeast and therefore allow human HSF1-dependent yeast growth. Our identification of these mutations, which are located in key hydrophobic residues of the amino-terminal and carboxyl-terminal leucine zippers, lend support to the hypothesis that these domains engage in coiled-coil interactions that repress the overall activity of the HSF1 protein and suggest that future modeling studies of this hypothetical interaction should consider these residues. Although the intra-molecular interaction between LZ1-3 and LZ4 has been widely accepted, definitive biochemical evidence of this interaction has not been reported. As such, it remains formally possible that these domains may repress HSF1 function by mediating a *trans*-acting interaction with other coiled-coil proteins. Because mutation of these coiled-coil domains results in the nuclear accumulation of the HSF1 trimer, we speculate that any such putative HSF1 interacting protein should be predominantly cytoplasmic, perhaps acting as a cytoplasmic anchor for HSF1. One candidate would be TRiC, a cytosolic chaperone complex that is conserved in yeast and mammalian cells which was previously shown to interact with a small molecule activator of human HSF1 trimerization and may be a tissue specific regulator of HSF1 in *C. elegans* (Neef *et al.* 2010; Guisbert *et al.* 2013). Alternatively, it remains formally possible that the coiled-coil regions of HSF1 mediate its repressive interactions with known HSF1-repressors such as Hsp70 and Hsp90. The hydrophobic nature of the coiled-coil domains makes this hypothesis potentially intriguing as protein chaperones often recognize exposed hydrophobic patches within proteins. Although the authors of previous studies have established the carboxyl-terminal activation domain of HSF1 as the primary binding site of Hsp70 (Abravaya *et al.* 1992; Shi *et al.* 1998), the specific HSF1 domains bound by Hsp70 or Hsp90 have not yet been identified.

Interestingly, although all of the mutations we identified in this screen result in the constitutive trimerization of human HSF1 both in yeast and mouse hsf1^−/−^ cells, as well as the nuclear localization of HSF1, these mutant HSF1 proteins are deficient in DNA binding until the cells are exposed to stress. These results were surprising as extensive evidence has shown that constitutively trimerized mammalian HSF1 mutants are competent to bind DNA *in vitro* using gel shift analysis (Rabindran *et al.* 1993; Zuo *et al.* 1994, 1995; Farkas *et al.* 1998). Although the precise defect underlying this inability to bind DNA remains unclear, it is possible that condensed chromatin architecture at specific loci precludes HSF1 DNA binding and an additional heat shock responsive regulatory event is required before trimerized HSF1 can access the promoter. Based on our data showing that a constitutively trimerized HSF1 protein is deficient in binding to the Hsp70 and Hsp25 promoter in the absence of stress, but able to bind to the IL-6 promoter, this hypothesis would predict that the Hsp70 promoter may have a very condensed chromatin architecture requiring chromatin remodeling factors for HSF1 DNA binding while HSF1 binding sites in the IL-6 promoter would be predicted to be unobstructed by chromatin and allow for unrestricted HSF1 binding even in the absence of stress. Unfortunately, detailed information describing the chromatin architecture of these promoters is lacking. Nevertheless, several previous reports suggest that condensed chromatin can preclude HSF1 DNA binding and the recruitment of many distinct chromatin remodeling factors is required for HSF1 DNA binding at the Hsp70 promoter *in vivo* (Becker *et al.* 1991; de La Serna *et al.* 2000; Badenhorst *et al.* 2002; Belova *et al.* 2008; Guertin *et al.* 2012). An alternative hypothesis to explain the differential binding of HSF1 to the IL-6 and Hsp70 promoters could be the architecture of the HSE. Although a consensus HSE (GAAnnTTCnnGAA) has been defined as the ideal high affinity-binding site for HSF1, many variations of this consensus sequence are bound by HSF1 at reduced affinities *in vivo*. Based on the constitutive binding of HSF1 to the IL-6 promoter but not to the Hsp70 promoter, one might predict that greater affinity HSEs are found in the IL-6 promoter compared with the Hsp70 promoter. However, the authors of a recent study have identified several putative HSEs in the IL-6 promoter, which vary significantly from the consensus HSE, and are bound by HSF1 at sevenfold lower affinity than the near-ideal HSE (GGAnnTTCnnGAC) found in the Hsp70 promoter (Inouye *et al.* 2007). As such, our observation that both wild-type HSF1 as well as the constitutively trimerized HSF1 mutants can bind constitutively to the IL-6 promoter but are unable to bind to the Hsp70 promoter supports the notion that binding of HSF1 to the Hsp70 promoter is being precluded by a compacted chromatin architecture.

Although the idea of loci-specific chromatin architecture dictating HSF1 DNA binding remains hypothetical, we propose that the stress responsive acquisition of DNA binding competency, at least in the context of the Hsp70 and Hsp25 promoter, serves as an additional level of regulation in the overall model of the HSF1 activation pathway. To this extent we report the identification of the HSF1 L189P mutant which despite its constitutively trimerized and nuclear state, remains largely defective in its ability to bind to the Hsp70 and Hsp25 promoters even upon heat stress stimulation and may in fact uncouple this additional layer of regulation. Because the L189P mutation is predicted to disrupt the carboxyl-terminal portion of the LZ3 domain, it is tempting to speculate that this portion of the trimerization domain may be indirectly important for HSF1 DNA binding *in vivo*. The importance of this domain being linked to DNA binding is striking as this small region of the protein is significantly removed from the DNA binding domain and is unlikely to make direct contact with the DNA. Although it is formally possible that the L189P mutation alters the overall structure of HSF1 in such a fashion that it affects the structure and function of the DNA binding domain, our findings that this mutant is fully functional in yeast, binds to the IL-6 promoter similar to the wild-type protein and is still able to provide some level of protection from cytotoxic stress suggests that this is not the case. However, it is difficult to effectively test this hypothesis without a crystal structure of HSF1 outside of the previously determined DNA binding domain in complex with DNA (Damberger *et al.* 1994, 1995; Littlefield and Nelson 1999). We favor an alternative explanation, in that the LZ3 domain is important for the recruitment of additional factors required for DNA binding in response to stress.

Previous evidence has shown that a significant group of additional factors assist in stress induced binding of HSF1 to the DNA. Several of these include the general transcription factor TFIID as well as the chromatin remodeling complexes NURF, Swi/Snf, ASC-2, HMGN1 and TAC1 (de La Serna *et al.* 2000; Badenhorst *et al.* 2002; Hong *et al.* 2004; Smith *et al.* 2004; Belova *et al.* 2008). The idea that LZ3 is important for recruitment of chromatin remodeling factors suggests that these factors might also have coiled-coil domains and that interactions between this domain and LZ3 mediates the stress responsive recruitment.
